# Too many crying babies: a systematic review of pain management practices during immunizations on YouTube

**DOI:** 10.1186/1471-2431-14-134

**Published:** 2014-05-29

**Authors:** Denise Harrison, Margaret Sampson, Jessica Reszel, Koowsar Abdulla, Nick Barrowman, Jordi Cumber, Ann Fuller, Claudia Li, Stuart Nicholls, Catherine M Pound

**Affiliations:** 1 Children’s Hospital of Eastern Ontario, 401 Smyth Road, Ottawa, ON K1H 8L1, Canada; 2 University of Ottawa, 451 Smyth Road, Ottawa, ON K1H 8M5, Canada; 3 Odette Cancer Centre, Sunnybrook Health Sciences Centre, 2075 Bayview Ave, Toronto, ON M4N 3M5, Canada

**Keywords:** YouTube, Infant, Immunization, Pain

## Abstract

**Background:**

Early childhood immunizations, although vital for preventative health, are painful and too often lead to fear of needles. Effective pain management strategies during infant immunizations include breastfeeding, sweet solutions, and upright front-to-front holding. However, it is unknown how often these strategies are used in clinical practice. We aimed to review the content of YouTube videos showing infants being immunized to ascertain parents’ and health care professionals’ use of pain management strategies, as well as to assess infants’ pain and distress.

**Methods:**

A systematic review of YouTube videos showing intramuscular injections in infants less than 12 months was completed using the search terms "baby injection" and "baby vaccine" to assess (1) the use of pain management strategies and (2) infant pain and distress. Pain was assessed by crying duration and pain scores using the FLACC (Face, Legs, Activity, Cry, Consolability) tool.

**Results:**

A total of 142 videos were included and coded by two trained individual viewers. Most infants received one injection (range of one to six). Almost all (94%) infants cried before or during the injections for a median of 33 seconds (IQR = 39), up to 146 seconds. FLACC scores during the immunizations were high, with a median of 10 (IQR = 3). No videos showed breastfeeding or the use of sucrose/sweet solutions during the injection(s), and only four (3%) videos showed the infants being held in a front-to-front position during the injections. Distraction using talking or singing was the most commonly used (66%) pain management strategy.

**Conclusions:**

YouTube videos of infants being immunized showed that infants were highly distressed during the procedures. There was no use of breastfeeding or sweet solutions and limited use of upright or front-to-front holding during the injections. This systematic review will be used as a baseline to evaluate the impact of future knowledge translation interventions using YouTube to improve pain management practices for infant immunizations.

## Background

Early childhood immunizations are essential for public health [1] however are painful, and often result in severe distress for infants and children [2,3]. They are also distressing for the parents [2,4-6]. Long-term risks of injections include fears of needle pain, parental non-adherence with immunization administration and avoidance of medical care [2,4,7-9]. It is therefore vital that evidence-based strategies be used to reduce immunization pain. This is especially important for infants, as untreated or poorly treated procedural pain in early infancy can lead to altered pain responses [10,11], and contribute to impaired brain development in preterm infants [12].

Extensive high quality evidence from large numbers of randomized controlled trials (RCTs) and systematic reviews demonstrate the analgesic effects of sweet solutions in newborn infants during commonly performed painful procedures [13-15], and in older infants during immunizations [16,17]. There is also sufficient evidence of pain-reducing effects of breastfeeding during immunizations [18-21], front-to-front upright holding [22] and some evidence of distraction in infants, especially when led by nurses/other clinicians [23]. These strategies are included in pain management recommendations in the Clinical Practice Guideline (CPG) "Reducing the pain of childhood vaccination" published in the Canadian Medical Association Journal [3] by the Help Eliminate Pain in KIDS (HELPinKIDS) team, the Immunize Canada website ( http://www.immunize.cpha.ca)*,* and in immunization guidelines internationally [24]. Despite such published recommendations, studies of pain management practices during immunization show that these strategies are rarely used [4,7,25,26], highlighting a gap between recommendations and clinical practice. In addition, when examining YouTube videos of infants being immunized, there is an abundance of videos showing a lack of utilization of pain management strategies.

Social media currently plays a large part in the way people communicate, and health information is one of the most frequently sought topics on the Internet [27]. Launched in December 2005, YouTube accounts for 60 percent of videos watched online [28] and the number of unique YouTube viewers per month is estimated at 136 million, three times the number of the next most popular video web site [29]. With such growing popularity, it is evident that YouTube potentially provides a new way to communicate evidence-based health information to a large number of people. As early childhood immunizations are a priority health topic that parents may explore on the internet, we examined YouTube immunization videos in order to establish what pain management strategies are used and the degree of distress infants are exhibiting.

In a previous review of YouTube immunization videos, 25% of the retrieved videos pertained to childhood immunizations. Of these, almost half conveyed negative messages about the painful nature of childhood immunizations [30]. However, to our knowledge, no studies have systematically examined the content of YouTube videos relating to childhood immunization pain or pain management practices. The purpose of this study was therefore to conduct a systematic review of YouTube videos showing infant immunizations, to ascertain the use of pain management strategies, and to assess infants’ pain and distress. This systematic review will be used as a baseline to evaluate the impact of knowledge translation interventions using YouTube to improve pain management practices.

## Methods

### Study design and screening

Systematic review of YouTube videos of infants receiving immunization injections.

### Search strategy

**Figure 1:**
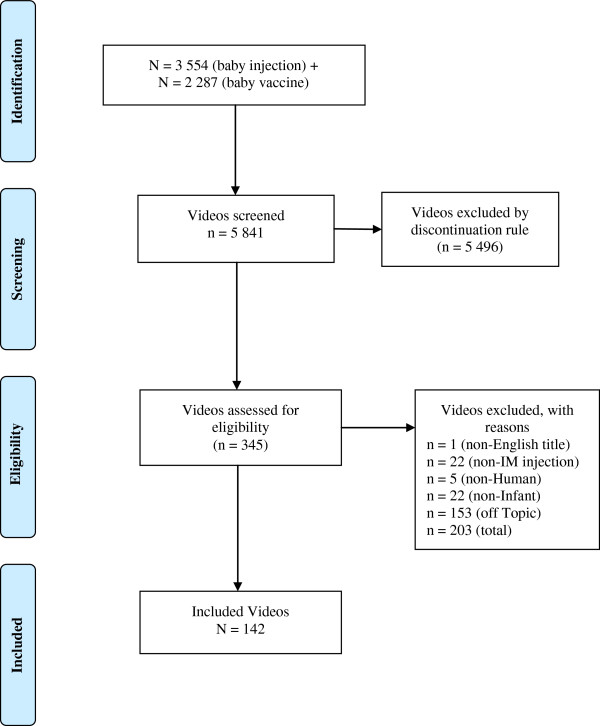
PRISMA diagram.

A preparatory review of YouTube search and review methods was done to inform decisions around the search and screening, such as screening order and discontinuation criteria [31]. In July 2012, and January 2013, a search of YouTube videos was completed using the default settings for the terms "baby injection" and "baby vaccine" as these were the two terms with the highest proportion of web searches, based on Google Trends [32]. The search and screening flow is shown in Figure 1.

A new YouTube account was used to eliminate the chance of search history influencing search rankings. The end point was determined through a discontinuation rule of 20 videos. When 20 consecutive videos did not meet eligibility criteria and were excluded, no further videos were screened. After viewing a video that fit the inclusion criteria, the researcher screened the first five related suggested videos that appeared [31].

As we aimed to review pain management practices used in actual clinical practice, we did not include educational videos or company/institution videos. Inclusion criteria included YouTube videos with English titles, available audio, portraying human infants 0 to 12 months of age (as assessed by the researcher) and showing at least one intramuscular injection. Videos that were educational or informational in nature were excluded.

### Data collection

Data collected for each included video comprised date of upload, number of views, age of infant (approximately 0, 2, 4, 6 or 12 months, as assessed by researcher), sex of infant (as per the data collectors judgment), number of injections, sex of main caregiver in the video, as well as the number of other people in the room, their sex and approximate age (child or adult), and the number of comments on each video. The first ten comments for each video were screened and classified into three categories: emotional, informational, or irrelevant. Lastly, YouTube allows viewers the option to click a "like" or "dislike" button to express their opinion on a video; the total number of "likes" and "dislikes" recorded underneath each video was recorded.

Each video was viewed for observable pain management strategies used during and after the injection. This included pharmacological, physical, and psychological strategies, such as topical anesthetics, positioning, distraction, breastfeeding, and the use of sweet solutions or sweet foods. To assess the infants’ pain and distress, we included crying incidence before and during the procedure, measured crying duration in seconds, and rated pain using the FLACC (Face, Legs, Activity, Cry, Consolability) tool [33]. The FLACC comprises 5 components, each with a possible value of 0 to 2, for a maximum total FLACC score of 10 indicating maximum pain. A primary coder completed FLACC scores on all included videos from the two searches in July 2012 and January 2013. To establish inter-rater agreement of FLACC scores for this project, we had a secondary coder independently view and score the first 92 (65%) videos included. Both coders were experienced pediatric nurses who received training on the FLACC scale.

### Data analysis

IBM SPSS [34] was used for all statistical analyses. Descriptive data are presented as means and standard deviations if normally distributed and presented as medians and interquartile ranges if non-normally distributed. Agreement between two individual coders on FLACC scores was assessed using intraclass correlation coefficient (ICC). If any one of the five components of a FLACC score was missing, the score was included and data was imputed using the calculation - (FLACC score/4) x 5. If two or more components were missing, the score for that time point was omitted from analysis.

### Ethics

This study was approved by the Children’s Hospital of Eastern Ontario (CHEO) Research Ethics Board in Ottawa, Canada (protocol #13/02X).

## Results

A total of 142 videos were included in the systematic review. The oldest included video was posted in February 2006. The median length of the videos was 74.5 seconds (IQR = 68, min = 10, max = 595) and the median number of views was 2,001 (IQR = 19,601, min = 1, max = 302,103). The included videos had a median of 1 like (IQR = 5, min = 0, max = 63) and a median of 0 dislikes (IQR = 1, min = 0, max = 42) at the time of initial viewing. The videos had a median of 1 comment (IQR = 7, min = 0, max = 476). Just over half (N = 78, 55%) of the videos were of infants receiving their 2-month immunizations. The majority of the caregivers were female (N = 97, 69%), while 28 (20%) were male and 16 (11%) were not visible at any time during the immunization video. In 38 (27%) videos, another adult and/or child were visible in the room. Seventy-six (54%) infants received one injection (median = 1, IQR = 1) with the number of injections ranging from one to six. The median length of procedure, defined as the time when the first injection site is cleansed to the time the last bandage or cotton swab is applied, was 34 seconds (IQR = 39), with a maximum of 256 seconds.

Pain management strategies during immunizations were evident in 72.5% of the videos. No videos showed use of breastfeeding, sucrose or other sweet solutions, or topical anesthetics. Eighty-eight (62%) infants were laid flat on their back during immunization and only four (3%) videos showed use of front-to-front upright holding. The most common pain management strategy observed was some form of distraction, with 66% of caregivers using singing or talking and 6% using a toy (Table 1).

**Table 1:** Observable pain management strategies used during immunization

**Strategy (n = 142)**	**N (%)**
Distraction using singing or talking*	93 (65.5)
Talking only	92 (64.8)
Singing and talking	1 (0.7)
Holding (any position)	54 (38)
Front-to-front	4 (2.8)
Front-to-back	21 (14.8)
Front-to-side	28 (19.7)
Combination of positions	1 (0.7)
Non-nutritive sucking	11 (7.7)
Distraction using any toy	9 (6.3)
Noisy toy	2 (1.4)
Silent toy	7 (4.9)
Rubbing at injection site	8 (5.6)

*2 missing – could not distinguish if singing or talking was used.

A total of 120 videos were assessed for pain management strategies after the completion of the injection. The remaining 22 videos ended immediately after the completion of the injection and were therefore excluded from this part of the analysis as pain management strategies after the immunization could not be assessed. Of the 120 videos, at least one observable pain management strategy post-immunization was evident in 96 (80%) videos. Distraction was the most common strategy used, with 80% of caregivers using singing or talking and 7% using a toy. Thirty-four (28%) videos showed front to front upright holding and 13 (9%) videos showed non-nutritive sucking after the immunization (Table 2).

**Table 2:** Observable pain management strategies used post immunization

**Strategy (n = 120)**	**N (%)**
Distraction using singing or talking*	94 (79.7)
Talking only	90 (76.3)
Singing and talking	4 (3.4)
Holding (any position)	65 (54.2)
Front-to-front	34 (28.3)
Front-to-back	7 (5.8)
Front-to-side	21 (17.5)
Combination of positions	3 (2.5)
Non-nutritive sucking	13 (9.2)
Distraction using any toy	10 (7.0)
Noisy toy	4 (2.8)
Silent toy	6 (4.2)

*2 missing – could not distinguish if singing or talking was used.

**Figure 2:**
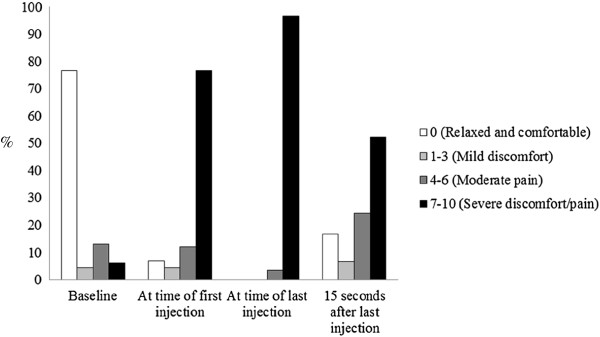
FLACC scores.

Fourteen (10%) videos showed infants crying before the procedure; all 14 of these infants continued to cry during the procedure. A total of 134 (94.4%) videos showed infants crying during the procedure. Of the 134 infants who cried, the median total cry time was 33 seconds (IQR = 39), with a maximum of 146 seconds.Infants’ pain during the immunizations was coded using the FLACC scores at four different time points: baseline (15 seconds before the first injection), at the time of the first injection, at the time of the last injection, and 15 seconds following the last injection (Figure 2). A total of 16 (11.3%) FLACC scores were imputed at baseline, 19 (13.4%) at time of first injection, 6 (4.25%) at time of last injection, and 27 (21.1%) fifteen seconds after the last injection. The most common missing component of the FLACC score at each time point was legs, followed by face. At baseline, the median FLACC score was 0 (n = 116, IQR = 0). During the injections, the majority of infants’ FLACC scores were the maximum score of 10. At the time of the first (n = 134, IQR = 3) and last injection (n = 61, IQR = 0) both median FLACC scores were 10, and the median FLACC score 15 seconds after the last injection was 7 (n = 107, IQR = 6.25) (Figure 2). The median FLACC scores of 10 at the time of first and last injection indicate high levels of pain.

Inter-rater agreement of the FLACC tool for the first 92 videos included (65% of all included videos) was established by two trained independent raters. Intraclass correlation coefficients (ICC) were 0.81 at baseline, 0.77 at time of first injection, and 0.76 at 15 seconds after the last injection, indicating acceptable agreement (Table 3).

**Table 3:** Inter-rater reliability

**Time point**	**N**	**ICC**	**95% CI**
Baseline	70	0.809	0.709–0.877
At time of first injection	85	0.774	0.672–0.847
15 seconds after last injection	79	0.756	0.643–0.837

## Discussion

To our knowledge, this is the first systematic review of videos posted on YouTube of infants undergoing immunization. The reasons for which parents post such videos of their infants undergoing immunizations are not known, and we did not seek to uncover motives for these posts. We aimed to conduct a systematic review of YouTube videos to gather evidence on the use of recommended pain management strategies, and to conduct pain assessments using crying duration and FLACC, the composite validated pain assessment tool. We found that most infants were highly distressed during the injections. This is disappointing given that strong evidence clearly shows the pain-reducing effects of breastfeeding infants [18-20,35], sweet solutions [17,36], using nurse/clinician-led distraction and upright holding [3,22,23] and given the work that has gone into translating this knowledge to the public and health care profession [3,37]. It is however possible that the pain control measures apparent during vaccinations as seen in the posted videos are not representative of all vaccinations, as the act of videotaping precludes holding the infant, breastfeeding or administering sucrose unless a tripod or third party is available to operate the camera. However, the observed infrequent use of recommended pain management strategies are concordant with results of published surveys of health care professionals’ pain management practices during immunization [4,7,25,26] as well as a recent observational study of pain management practices in infants during immunization [38]. This highlights that, so far, current evidence and guidelines relating to pain management strategies, most of which has been available prior to the date of the first YouTube posting, have been unsuccessful in changing immunization pain practices. It is not known whether this is an issue of *reach*, and therefore a lack of knowledge concerning best pain management practices, or whether this information is *known*, but not used by the multitude of different groups of immunizers and parents of infants. However, it is impossible to know when parents produced the videos and it is possible that some of the videos were filmed prior to the publication and dissemination of recommended evidence-based pain management practices. Regardless, a state of play exists currently where information is known, but is inconsistently used in clinical practice [4,7,25,26,38].

Taddio et al. attributed suboptimal pain management during childhood immunization to lack of parental knowledge about pain, health care professional attitudes to pain severity and effective pain management, and societal attitudes about pain including dismissing the impact of needle pain [4]. Taddio et al. also presented a number of myths concerning barriers to using effective pain management strategies for infants. Myths concerning using breastfeeding for pain management include beliefs that the infants will choke, or associate the mother with pain and myths concerning sucrose include interfering with breastfeeding and damaging infants’ teeth. Myths concerning the need to provide pain management strategies include that infants cry anyway; they need to cope with pain; and they get used to shots (Pages S160- S161 [4]). Pillai-Riddel et al. suggests that despite the knowledge health care professionals have about short-term pain and distress-reducing benefits of strategies such as breastfeeding and sweet solutions, they may not believe that putting these pain-reducing strategies into place is a clinical priority, as there is little known about the long term benefits of reducing childhood immunization pain [39]. This belief exists in the face of clear and extensive descriptions about high levels of distress infants exhibit during immunizations [38,40] and the fact that parents may avoid having their children immunized due to concerns about pain [9]. Additional barriers may be due to availability of commercially manufactured sucrose in diverse settings where immunizations take place and no knowledge to make home-made sucrose; cost factors such as purchase cost of topical anesthetics; organizational factors such as privacy for breastfeeding, or perceived increased ease of vaccinating if the infant is lying on an examination table as opposed to being held.

Although our findings of high levels of distress may be influenced by the proportionally larger number of videos showing 2-month old infants, who may exhibit higher levels of distress than older infants [41], infants of all ages can become distressed during immunizations [38,40]. There is a high prevalence of fear of needles in children, which could likely have developed as a result of the painful injections in infancy [2,4,7,8,42]. These concerning factors highlight the need for health care professionals and parents of infants and young children to work together to reduce the pain of childhood vaccinations. Although our results, consistent with previous research, show that parental talking, singing and reassurance, is commonly used (for example, talking or singing was observed in 66% the videos), and most parents instinctively use reassurance, this has been shown to lead to higher exhibition of pain behaviors [38]. This may also account for the high levels of distress as observed in the majority of infants in this study, highlighting that health care professionals need to support parents to provide effective pain management strategies.

YouTube may be a promising medium for disseminating knowledge to health care professionals and parents. The YouTube website attracts over 2 billion views daily [43], and its use as a knowledge translation forum for researchers and health professionals is growing in popularity. In this systematic review of YouTube videos of infant immunizations, we used YouTube as the source of information to study – i.e., the ‘participants’ are the posted YouTube videos. Other topics relating to pediatric health care that have been researched using YouTube include information on the management of burn injuries [43], information on tonsillectomy [44], and dental fear and anxiety [45]. Topics researched using YouTube in adults include concussion [46], inflammatory bowel disease [47] and anorexia [48]. To facilitate the use of YouTube as an information source, Sampson et al. published a review on the methods used to undertake reviews of YouTube [31,49].

One example of health care professionals using YouTube as a medium for information sharing with consumers is the Canadian Institutes of Health Research (CIHR) funded HELPinKIDS team’s utilization of YouTube for disseminating knowledge about effective pain management strategies for infants during childhood immunizations. In 2012 the team posted a comprehensive educational YouTube video discussing a variety of pain management strategies, including the use of breastfeeding, sucrose and secure front-to-front holding (HELPinKIDS Managing Infant Pain https://www.youtube.com/watch?v=jxnDc2PxGUc&list=PLJH3y0duq2ZEQ_KkfKVkcLwZUk3HPV6xj&index=1). The video is over 8 minutes in duration, considerably longer than the typical videos posted on YouTube by parents. Since posting to YouTube in November 2012, the video had 4,869 hits in 12 months (as of November, 13, 2013). The impact of this teaching video is not yet known. However, compared to some other YouTube videos showing infants vigorously crying during their injections, the HELPinKIDS educational video has had much fewer hits, highlighting that attention seems to be drawn to the consumer posted videos showing crying infants, rather than the instructional video highlighting how to reduce pain during immunizations. This highlights the need for health care professionals and researchers wishing to utilize YouTube as a knowledge translation and dissemination tool to understand the most effective ways to ensure popularity, when practice change is a goal.

### Limitations

There are several limitations to this study. Consumers posted all videos with no pre-set standards for rigor or quality. A systematic review of such varying quality videos is therefore acknowledged to be less rigorous than a systematic review of published RCTs. Not all videos were of sufficient quality for analysis. For example, in 26 videos, we were not able to sufficiently see the infants to determine FLACC scores at baseline, and, in eight videos, we could not determine FLACC scores at the time of first injection. In 22 cases, pain management strategies used after completion of the injection could not be visualized due to the video footage ending as soon as the injections were completed. Furthermore, it was often impossible to determine if the vaccine administration technique and the order of vaccine administration was in accordance with current recommended guidelines (i.e. rapid injection technique with no aspiration and most painful injection administered last) [3], which are known to impact pain responses [50,51]. In addition, as stated above, it is impossible to know when the videos were filmed, and it is possible that some videos may have been produced years prior to the availability of knowledge translation products and recommended evidence-based pain management practices.

Another limitation in conducting a systematic review of consumer posted videos on YouTube relates to the risk of ‘posting’ bias. The pain management strategies used in the reviewed videos may not be representative of all vaccinations for two reasons. First, as previously discussed, the act of videotaping precludes the use of pain management strategies such as holding the infant, breastfeeding or administering sucrose unless a tripod or third party is available to operate the camera. Second, caregivers who used pain management strategies, most notably breastfeeding, may not be comfortable choosing to have the procedure video recorded and posted publicly.

## Conclusions

In conclusion, this systematic review of 142 YouTube videos showing infants being immunized highlights that most infants were highly distressed during the injections; there was no evidence of use of breastfeeding or sweet solutions and infants were rarely securely held in an upright front-to-front position. This systematic review of YouTube videos of infant immunization will be used as a baseline to evaluate the impact of a knowledge translation intervention using YouTube, aimed at improving pain management practices.

## Abbreviations

CIHR: Canadian Institutes of Health Research; CPG: Clinical practice guideline; ICC: Intraclass correlation coefficient; FLACC: Face, Legs, Activity, Cry, Consolability; RCT: Randomized controlled trial.

## Competing interests

The authors have no competing interests to disclose.

## Authors’ contributions

DH, MS, NB, JC, AF, CL, SN and CP contributed to the conception and design of the study; KA and JC conducted the data collection; JR and NB contributed to the data analysis; DH and JR participated in the writing of the manuscript; all authors reviewed and approved the final manuscript.

## Authors’ information

DH is the Chair in Nursing Care of Children Youth and Families at the Children’s Hospital of Eastern Ontario (CHEO) and the University of Ottawa; MS is the Manager of Library Services at CHEO; JR is a research coordinator at CHEO Research Institute (RI); KA is a research assistant and Registered Nurse in the neonatal intensive care unit at CHEO; NB is a Senior Biostatistician at CHEO RI; JC is a research assistant at CHEO and a Registered Nurse in labour and delivery at The Ottawa Hospital; AF is the Director of Public Relations at CHEO; CL is now a Clinical Research Associate at the Odette Cancer Centre – Sunnybrook Health Sciences Centre but was working for the CHEO RI at the time of this study; SN is a post-doctoral fellow and research associate at the University of Ottawa; CP is a pediatrician at CHEO.

## Pre-publication history

The pre-publication history for this paper can be accessed here:

http://www.biomedcentral.com/1471-2431/14/134/prepub

## References

[B1] Briss PA Rodewald LE Hinman AR Shefer AM Strikas RA Bernier RR Carande-Kulis VG Yusuf HR Ndiaye SM Williams SM Reviews of evidence regarding interventions to improve vaccination coverage in children, adolescents, and adults. The Task Force on Community Preventive Services Am J Prev Med 2000 18 1 Suppl 97 140 10806982 10.1016/s0749-3797(99)00118-x

[B2] Schechter NL Zempsky WT Cohen LL McGrath PJ McMurtry CM Bright NS Pain reduction during pediatric immunizations: evidence-based review and recommendations Pediatrics 2007 119 5 e1184 e1198 10.1542/peds.2006-1107 17473085

[B3] Taddio A Appleton M Bortolussi R Chambers C Dubey V Halperin S Hanrahan A Ipp M Lockett D MacDonald N Midmer D Mousmanis P Palda V Pielak K Pillai Riddell R Rieder M Scott J Shah V Reducing the pain of childhood vaccination: an evidence-based clinical practice guideline CMAJ 2010 182 18 E843 E855 10.1503/cmaj.101720 21098062 PMC3001531

[B4] Taddio A Chambers CT Halperin SA Ipp M Lockett D Rieder MJ Shah V Inadequate pain management during routine childhood immunizations: the nerve of it Clin Ther 2009 31 Suppl 2 S152 S167 19781434 10.1016/j.clinthera.2009.07.022

[B5] Parvez E Stinson J Boon H Goldman J Shah V Taddio A Mothers' beliefs about analgesia during childhood immunization Paediatr Child Health 2010 15 5 289 293 21532793 10.1093/pch/15.5.289 PMC2912630

[B6] Mills E Jadad AR Ross C Wilson K Systematic review of qualitative studies exploring parental beliefs and attitudes toward childhood vaccination identifies common barriers to vaccination J Clin Epidemiol 2005 58 11 1081 1088 10.1016/j.jclinepi.2005.09.002 16223649

[B7] Taddio A Manley J Potash L Ipp M Sgro M Shah V Routine immunization practices: use of topical anesthetics and oral analgesics Pediatrics 2007 120 3 e637 e643 10.1542/peds.2006-3351 17766503

[B8] Wright S Yelland M Heathcote K Ng S-K Wright G Fear of needles–nature and prevalence in general practice Aust Fam Physician 2009 38 3 172 176 19283260

[B9] Diekema DS Improving childhood vaccination rates N Engl J Med 2012 366 5 391 393 10.1056/NEJMp1113008 22296072

[B10] Taddio A Katz J The effects of early pain experience in neonates on pain responses in infancy and childhood Paediatr Drugs 2005 7 4 245 257 10.2165/00148581-200507040-00004 16117561

[B11] Taddio A Katz J Ilersich AL Koren G Effect of neonatal circumcision on pain response during subsequent routine vaccination Lancet 1997 349 9052 599 603 10.1016/S0140-6736(96)10316-0 9057731

[B12] Brummelte S Grunau RE Chau V Poskitt KJ Brant R Vinall J Gover A Synnes AR Miller SP Procedural pain and brain development in premature newborns Ann Neurol 2012 71 3 385 396 10.1002/ana.22267 22374882 PMC3760843

[B13] Harrison D Bueno M Yamada J Adams-Webber T Stevens B Analgesic effects of sweet tasting solutions in infants: Do we have equipoise yet? Pediatrics 2010 126 5 894 902 10.1542/peds.2010-1593 20937658

[B14] Stevens B Yamada J Ohlsson A Sucrose for analgesia in newborn infants undergoing painful procedures Cochrane Database Syst Rev 2013 Issue 1. Art. No.: CD001069 doi:10.1002/14651858.CD001069.pub4 10.1002/14651858.CD001069.pub4 23440783

[B15] Bueno M Yamada J Harrison D Kahn S Adams-Webber T Beyene J Ohlsson A Stevens B A systematic review and meta-analyses of non-sucrose sweet solutions sucrose for pain relief in neonates Pain Res Manag 2013 18 3 153 161 23748256 10.1155/2013/956549 PMC3673933

[B16] Harrison D Stevens B Bueno M Yamada J Adams-Webber T Beyene J Ohlsson A Efficacy of sweet solutions for analgesia in infants between 1 and 12 months of age: a systematic review Arch Dis Child 2010 95 6 406 413 10.1136/adc.2009.174227 20463370

[B17] Kassab M Foster JP Foureur M Fowler C Sweet-tasting solutions for needle-related procedural pain in infants one month to one year of age Cochrane Database Syst Rev 2012 Issue 3 Art. No.: CD008411 doi:10.1002/14651858.CD008411 10.1002/14651858.CD008411.pub2 PMC6369933 23235662

[B18] Efe E Ozer ZC The use of breast-feeding for pain relief during neonatal immunization injections Appl Nurs Res 2007 20 1 10 16 10.1016/j.apnr.2005.10.005 17259038

[B19] Dilli D Küçük I Dallar Y Interventions to reduce pain during vaccination in infancy J Pediatr 2009 154 3 385 390 10.1016/j.jpeds.2008.08.037 18849052

[B20] Razek AA El-Dein AN Effect of breast-feeding on pain relief during infant immunization injections Int J Nurs Pract 2009 15 2 99 104 10.1111/j.1440-172X.2009.01728.x 19335527

[B21] Shah V Taddio A Rieder MJ Effectiveness and tolerability of pharmacologic and combined interventions for reducing injection pain during routine childhood immunizations: systematic review and meta-analyses Clin Ther 2009 31 SUPPL. 2 S104 S151 19781433 10.1016/j.clinthera.2009.08.001

[B22] Taddio A Ilersich AL Ipp M Kikuta A Shah V HELPinKIDS Team Physical interventions and injection techniques for reducing injection pain during routine childhood immunizations: systematic review of randomized controlled trials and quasi-randomized controlled trials Clin Ther 2009 31 Suppl 2 S48 S76 19781436 10.1016/j.clinthera.2009.07.024

[B23] Chambers CT Taddio A Uman LS McMurtry CM Team H Psychological interventions for reducing pain and distress during routine childhood immunizations: a systematic review Clin Ther 2009 31 Suppl 2 S77 S103 19781437 10.1016/j.clinthera.2009.07.023

[B24] Australian Government The Australian Immunisation Handbook 10th Edition Department of Health and Ageing, National Health and Medical Research Council 2013 9

[B25] Harrison D Elia S Royle J Manias E Pain management strategies used during early childhood immunisation in Victoria J Paediatr Child Health 2013 49 313 318 10.1111/jpc.12161 23489548

[B26] Schechter NL Bernstein BA Zempsky WT Bright NS Willard AK Educational outreach to reduce immunization pain in office settings Pediatrics 2010 126 6 e1514 e1521 10.1542/peds.2010-1597 21078736

[B27] McMullan M Patients using the internet to obtain health information: How this affects the patient-health professional relationship Patient Educ Couns 2005 63 1–2 24 28 16406474 10.1016/j.pec.2005.10.006

[B28] English K Sweetser KD Ancu M YouTube-ification of political talk: an examination of persuasion appeals in viral video Am Behav Sci 2011 55 6 733 748 10.1177/0002764211398090

[B29] Top 10 U.S. Online Video Sites, Total http://nielsen.com/us/en/insights/top10s/internet.html

[B30] Keelan J Pavri-Garcia V Tomlinson G Wilson K YouTube as a source of information on immunization: a content analysis JAMA 2007 298 21 2482 2484 18056901 10.1001/jama.298.21.2482

[B31] Sampson M Cumber J Joly C Li C Fuller A Pound C Harrison D A systematic review of methods for studying consumer health YouTube videos, with implications for systematic reviews PeerJ 2013 1 e147 24058879 10.7717/peerj.147 PMC3775625

[B32] Google Google Trends - About [ http://www.google.com/intl/en/trends/about.html]

[B33] Merkel SI Voepel-Lewis T Shayevitz JR Malviya S Practice applications of research. The FLACC: a behavioral scale for scoring postoperative pain in young children Pediatr Nurs 1997 23 3 293 297 9220806

[B34] Corp IBM IBM SPSS Statistics for Windows, Version 20.0 2011 Armonk, NY: IBM Corp

[B35] Harrison D Yamada J Stevens B Strategies for the prevention and management of neonatal and infant pain Curr Pain Headache Rep 2010 14 113 123 10.1007/s11916-009-0091-0 20425200

[B36] Harrison D Yamada J Adams-Webber T Ohlsson A Beyene J Stevens B Sweet tasting solutions for needle-related procedural pain in infants and children aged 1 to 16 years Cochrane Database Syst Rev 2011 10 Art. No.: CD008408 doi:10.1002/14651858.CD008408.pub2 10.1002/14651858.CD008408.pub2 21975781

[B37] Taddio A Shah V Leung E Wang J Parikh C Smart S Hetherington R Ipp M Riddell RP Sgro M Jovicic A Franck L Knowledge translation of the HELPinKIDS clinical practice guideline for managing childhood vaccination pain: usability and knowledge uptake of educational materials directed to new parents BMC Pediatr 2013 13 23 10.1186/1471-2431-13-23 23394070 PMC3585914

[B38] Lisi D Campbell L Pillai Riddell R Garfield H Greenberg S Naturalistic parental pain management during immunizations over the first year of life: observational norms from the OUCH cohort Pain 2013 154 July 1245 1253 23726370 10.1016/j.pain.2013.03.036

[B39] Pillai Riddell R Response to letter to Naturalistic studies of procedural pain management in infants – Is it ethical to not provide pain management? Pain 2013 154 1896 1897 10.1016/j.pain.2013.05.027 23711483

[B40] Harrison D Elia S Royle J Barrowman N Sucrose and lollypops to reduce immunisation pain in toddlers and young children: Two pilot randomised controlled trials Int J Nurs Stud 2014 17 1 20 28

[B41] Ipp M Taddio A Goldbach M Ben David S Stevens B Koren G Effects of age, gender and holding on pain response during infant immunization Can J Clin Pharmacol 2004 11 1 e2 e7 15226521

[B42] Rennick JE McHarg LF Dell’Api M Johnston CC Stevens B Developing the Children's Critical Illness Impact Scale: capturing stories from children, parents, and staff Pediatr Crit Care Med 2008 9 3 252 260 10.1097/PCC.0b013e31816c70d4 18446107

[B43] Oomman A Sarwar U Javed M Hemington-Gorse S YouTube as a potential online source of information in the prevention and management of paediatric burn injuries Burns 2013 39 8 1652 10.1016/j.burns.2013.06.012 23876783

[B44] Strychnine JE Nayan S Farrokhyar F Maclean J YouTube: a good source of information on pediatric tonsillectomy? Int J Pediatr Otorhinolaryngol 2013 77 6 972 975 10.1016/j.ijporl.2013.03.023 23598152

[B45] Gao X Hamzah SH Yiu CKY McGrath C King NM Dental fear and anxiety in children and adolescents: qualitative study using YouTube J Med Internet Res 2013 15 2 e29 10.2196/jmir.2290 23435094 PMC3636260

[B46] Williams D Sullivan J Schneiders AG Ahmed OH Lee H Balasundaram AP McCrory PR Big hits on the small screen: an evaluation of concussion-related videos on YouTube Br J Sports Med 2013 48 2 1 5 10.1136/bjsports-2012-091853 23446643

[B47] Fortinsky KJ Fournier MR Benchimol EI Internet and electronic resources for inflammatory bowel disease: a primer for providers and patients Inflamm Bowel Dis 2012 18 6 1156 1163 10.1002/ibd.22834 22147497

[B48] Syed-Abdul S Fernandez-Luque L Jian WS Li YC Crain S Hsu MH Wang YC Khandregzen D Chuluunbaatar E Nguyen PA Liou DM Misleading health-related information promoted through video-based social media: anorexia on YouTube J Med Internet Res 2013 15 2 e30 10.2196/jmir.2237 23406655 PMC3636813

[B49] Sampson M Cumber J Joly C Li C Fuller A Pound C Harrison D So many screaming babies: an informal methods review of studies of consumer health youtube videos Building Bridges: UNYOC/OVHLA 2012 Joint Conference 2012 Cornwall, Ontario, Canada

[B50] Ipp M Parkin PC Lear N Goldbach M Taddio A Order of vaccine injection and infant pain response Arch Pediatr Adolesc Med 2009 163 5 469 472 10.1001/archpediatrics.2009.35 19414694

[B51] Ipp M Taddio A Sam J Gladbach M Parkin PC Vaccine-related pain: randomised controlled trial of two injection techniques Arch Dis Child 2007 92 12 1105 1108 10.1136/adc.2007.118695 17686797 PMC2066084

